# Generation and Characterisation of a Reference Transcriptome for Lentil (*Lens culinaris* Medik.)

**DOI:** 10.3390/ijms17111887

**Published:** 2016-11-12

**Authors:** Shimna Sudheesh, Preeti Verma, John W. Forster, Noel O. I. Cogan, Sukhjiwan Kaur

**Affiliations:** 1 Biosciences Research, Agriculture Victoria, AgriBio, 5 Ring Road, La Trobe University, Bundoora, VIC 3083, Australia; shimna.sudheesh@ecodev.vic.gov.au (S.S.); preeti.verma@ecodev.vic.gov.au (P.V.); john.forster@ecodev.vic.gov.au (J.W.F.); noel.cogan@ecodev.vic.gov.au (N.O.I.C.); 2 School of Applied Systems Biology, La Trobe University, Bundoora, VIC 3086, Australia

**Keywords:** legume, pulse, Illumina, de novo assembly, sequence annotation, tissue-specific gene expression

## Abstract

RNA-Seq using second-generation sequencing technologies permits generation of a reference unigene set for a given species, in the absence of a well-annotated genome sequence, supporting functional genomics studies, gene characterisation and detailed expression analysis for specific morphophysiological or environmental stress response traits. A reference unigene set for lentil has been developed, consisting of 58,986 contigs and scaffolds with an N50 length of 1719 bp. Comparison to gene complements from related species, reference protein databases, previously published lentil transcriptomes and a draft genome sequence validated the current dataset in terms of degree of completeness and utility. A large proportion (98%) of unigenes were expressed in more than one tissue, at varying levels. Candidate genes associated with mechanisms of tolerance to both boron toxicity and time of flowering were identified, which can eventually be used for the development of gene-based markers. This study has provided a comprehensive, assembled and annotated reference gene set for lentil that can be used for multiple applications, permitting identification of genes for pathway-specific expression analysis, genetic modification approaches, development of resources for genotypic analysis, and assistance in the annotation of a future lentil genome sequence.

## 1. Introduction

The application of next-generation sequencing (NGS) technologies to sequence mRNA (RNA-Seq) can provide comprehensive characterisation of the gene-space of a given organism, allowing definition of an extensive gene catalogue, identification of specific coding DNA sequences (CDSs), development of gene-associated genetic markers, comparative genomics analysis and quantification of gene expression [1]. Regular advances in NGS technologies have removed the impediments to characterisation of the transcriptomes and genomes of crop species with relatively lower scales of cultivation and economic value, such as lentil (*Lens culinaris* Medik.) [2,3,4]. Lentil is grown in approximately 70 countries and consumed in more than 120, with an annual production of ca. 4 Mt [5]. Archaeological studies have confirmed the presence of lentils in the northern part of the Fertile Crescent (Turkey, Syria, Iraq) from 8500–6000 BC [6] and the species is believed to have been first domesticated in south-west Asia from the latter part of the sixth millennium BC [7,8]. Lentils provide a rich and inexpensive source of protein, carbohydrates, micronutrients and vitamins and are a major source of nutrition in developing nations. As a legume, lentils play a beneficial role in agriculture through nitrogen fixation and can also assist in management of weeds and pathogen through crop rotation. From an economic perspective, the crop generates an income for small-scale farmers in the dryland agricultural ecosystems of South Asia, Sub-Saharan Africa, West Asia, and North Africa [9,10].

Lentil is a diploid (2*n* = 2*x* = 14), annual self-pollinating crop with a haploid genome size of ca. 4 Gbp [10,11]. An international genome sequencing effort to deliver the reference lentil genome is currently underway, leading to the recent release of an initial draft genome assembly from the cultivar CDC Redberry [12]. However, this assembly is still in a preliminary form, with minimal gene annotation and limited access [13]. Additional data analysis is in progress to further improve the genome assembly and deliver an annotated lentil reference genome in the near future. Whole-genome sequencing represents the most comprehensive strategy to deliver molecular tools and resources to assist crop improvement, but is still a costly and complex exercise when applied to plant genomes of moderate to large sizes. Over a thousand-fold variation in genome sequence is observed across the plant kingdom [14]. Species with large nuclear genomes, such as lentil, field pea, faba bean (among cool-season legumes) and wheat, maize and barley (among other groups) contain a substantial quantity of repetitive DNA (approx. 50%–70% of the nuclear genome complement), largely composed of different classes of retrotransposons and repeat elements. These repetitive DNA components generate difficulties for genome assembly. However, for many applications, analysis of the genic portion of the genome is sufficient to provide information relevant to crop improvement and production. A number of methods are available for sampling of the gene space, including enrichment for demethylated regions of the genome [15], and use of reassociation kinetics to obtain low copy sequences [16]. However, the most pragmatic approach is through direct sampling of the transcriptome, which can then deliver functionally associated gene-based markers for the purpose of breeding activities [17,18].

NGS methods offer a cost-effective means to access the gene space of a target organism through in-depth sequencing of the genome and transcriptome [17,19,20]. A large number of transcriptome studies have been performed on a diverse array of organisms, through the use of either microarrays or, more recently RNA-Seq [21,22], to identify genes controlling pathways that underlie various biological processes [23,24,25,26]. High-throughput sequencing approaches have rendered microarrays obsolete, as transcriptome sequencing can deliver more direct quantitative data on gene expression, as well as the added value of detecting novel transcripts and isoforms, defining exon/intron boundaries, and revealing sequence polymorphisms and splice variants [27]. In addition, as an “open” system, RNA-Seq is capable of detecting transcripts that are not represented on “closed” microarray systems, as well as delivering a more accurate dynamic range of quantification for gene expression.

Analysis of completed plant genomes, both of model and crop species, identified a range of transcriptome size for diploid species that varies from 50 to 80 Mb [28,29]. The average gene length (without 5′- and 3′-untranslated regions [UTRs]) and gene number for these completed references varies from 1060 and 25,532 bp (*Medicago truncatula* Gaertn.) to 2956 and 62,388 bp (*Brachypodium distachyon* L.). As plant transcriptomes display considerably lower variation in total size than the respective genomes, de novo generation of a unigene reference for an otherwise relatively underdeveloped species is tractable through RNA-Seq approaches with current sequencing technology and assembly software.

De novo assembly of RNA-Seq-derived data allows identification of the overwhelming majority of expressed genes without the need for a reference genome sequence. RNA-Seq datasets have been recently produced for several crop species, including legumes such as pea (*Pisum sativum* L.) [18,30,31], lentil [8,17,32], chickpea (*Cicer arietinum* L.) [24,33], soybean (*Glycine max* [L.] Merr.) [23], common bean (*Phaseolus vulgaris* L.) [26], pigeon pea (*Cajanus cajan* [L.] Millsp.) [34] and faba bean (*Vicia faba* L.) [18,35]. However, the majority of these datasets were not intended to generate a comprehensive reference unigene set, but instead to support gene-based marker discovery for assessment of genetic diversity, linkage mapping and trait dissection. A comprehensive unigene set offers a broad range of opportunities for extensive downstream data analysis, such as determination of transcript expression patterns, identification of uncharacterised genes with specific patterns of expression, characterisation of the genetic basis of key metabolic pathways and discovery of sequence polymorphisms for genome-wide association studies [22].

Integration of transcriptome profiling with genetic linkage mapping provides a promising tool for the identification of candidate genes and allelic sequence variants responsible for simple, or even complex traits. Such an integrated genomic approach was successfully applied in the identification of candidate genes governing plant height and agro-morphological traits in chickpea [36,37]. In lentil, a limited number of SNP or gene based linkage maps have been published [8,38,39] and used for quantitative trait loci (QTL) identification [38,39]. QTLs or linked markers associated with traits such as boron (B) toxicity tolerance, flowering time (FT) and seed characteristics have been identified [38,39]. A SNP and SSR-based linkage map of an intraspecific mapping population (Cassab × ILL2024) was developed, facilitating the identification of genomic regions and candidate genes associated with B toxicity tolerance. In the absence of a genome or comprehensive transcriptome assembly, a synteny-based approach with other plant species was used to identify candidate genes associated with B toxicity tolerance [38]. Identification of a broader range of candidate genes from a transcriptome assembly, when combined with genetic analysis, could extend the scope of such a study and assist the development of diagnostic genetic markers. FT is another important trait that can influence the yield of lentil in environments characterised by long day-lengths and a short growing period. At different latitudes, climatic regions, seasons and altitudes, plants exhibit differing responses to environmental stimuli that induce flowering [40]. Several quantitative trait loci (QTLs) for natural variation of FT have been reported [39,41,42]. As for boron toxicity tolerance, interrogation of the assembly could assist the identification of plausible candidate genes.

In the present study, a comprehensive unigene reference transcriptome was developed for the lentil cultivar Cassab. This cultivar was used early on in lentil breeding and to develop recombinant inbred lines (RILs) for a number of trait dissection studies, therefore selected for deep transcriptome analysis to enable genomic wide association studies in lentil. Sequence reads from multiple cDNA libraries were combined and de novo assembled, followed by comparison to the genic complements of related species, sequence annotation and assessment of tissue-specific expression. Identification of candidate genes for B toxicity tolerance and flowering time demonstrated the value of the dataset for the interpretation of biological processes. The unigene set will provide a resource for the development of tools for molecular breeding of lentil, as well as annotation of the current and future reference genome sequence.

## 2. Results

### 2.1. De Novo Sequence Assembly

A total of 7 RNA-Seq libraries were generated and sequenced from a variety of tissue types of cultivar Cassab. The raw sequence data was then filtered to remove adaptor sequences and exclude low quality or short reads, resulting in a high quality set of over 660 million paired-end reads, with an average read length of 120 bp. Details of the filtered sequences for each tissue-specific library are provided in Table S1. An average of 94 million reads were available for each individual tissue type. The filtered sequences were then assembled, and after empirical testing the *k*-mer size of 91 was found to be optimal using 84% of the reads and generating 107,311 contigs, with N50 of 836 bp. These assembled contigs were then processed by paired-end joining and gap filling, to combine into 77,778 transcripts (including both scaffolds and contigs), representing a cumulative length of 76.9 Mbp, with an N50 of 1731 bp (Table 1). The set of scaffolds that were identified as specific loci and contained multiple sequence entries described as forks, bubbles or complex, were further analysed and assembled through the use of the CAP3 program. In addition, a total of 15,535 transcripts that were shorter than a pair of sequence reads (240 bp) were removed. The result of the extensive assembly and filtering was a total of 58,994 transcripts (total assembly length—66,767,914 bp), with an N50 length of 1719 bp (Table 1). The average transcript size was 1132 bp, the longest being 21,632 bp. The final assembly contained a substantial number of large transcripts, 38,430 (64%) > 500 bp, 24,932 (42%) > 1000 bp and 9236 (16%) > 2000 bp (Figure S1).

### 2.2. Functional Annotation and Classification

**Figure 1:**
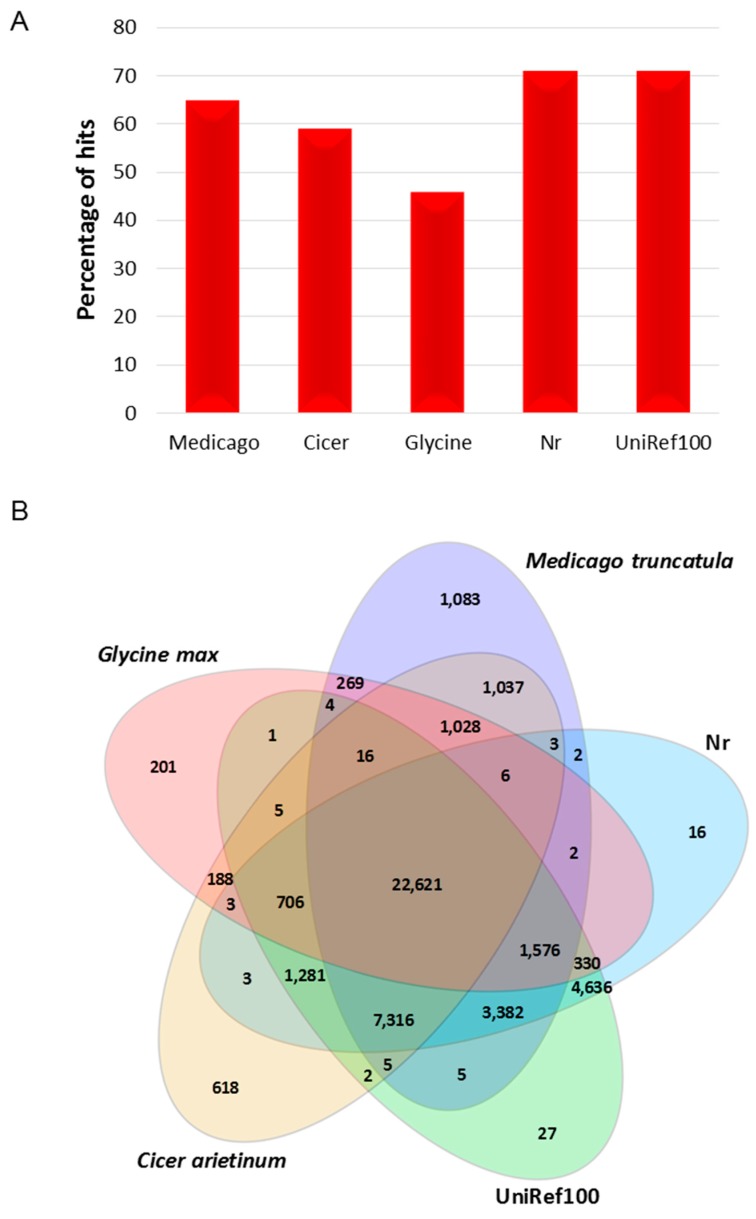
Sequence conservation of the Cassab-derived reference unigene transcriptome in comparison to sequences from other species: (**A**) percentage of sequence similarity of Cassab-derived reference transcripts with sequences from other plant species in the Nr and UniRef100 databases; (**B**) Venn diagram summarising the distribution of BLAST matches between the Cassab-derived reference unigene transcriptome and sequences from three other legume genomes and two databases. Numbers within the Venn diagram indicate the number of sequences sharing similarity using BLAST.

For validation and annotation of the reference transcriptome assembly, all transcripts were BLASTX analysed against the non-redundant (Nr) and UniRef100 databases, allowing identification of a total of 41,949 transcripts (71%) with significantly similar proteins (41,883 and 41,913, respectively) (Figure 1, Table S2). Of these transcripts, 41,849 (99.7%) identified common proteins between both databases, while 100 transcripts showed similarity to a single database. A total of 41,844 transcripts showed matches to plant proteins, representing 27,396 and 27,425 unique annotations from Nr and UniRef100 databases respectively, while the remaining 105 transcripts showed highest matches of moderate similarity to non-plant-derived sequences. However, these anomalies were resolved after BLASTN searches to CDS datasets of closely related legume species and lentil draft genome assembly v0.8 (accessed through organisational participation in the international lentil genome sequencing effort), that identified a majority of transcripts with a higher match to these sources. A final set of only 8 non-plant-derived sequences were removed from the reference unigene set. The length of the annotated transcripts varied from 241 to 21,632 bp, with an average of 1371 bp. A total of 32,224 (77%) annotated (Nr) transcripts were ≥500 bp, in which 23,170 transcripts were longer than 1000 bp in size (Figure S1). The distribution of annotations based on BLASTX analysis exhibited the highest number of matches against sequences of *M. truncatula*, followed by chickpea, and then other plant protein sequences within the Nr database of NCBI. The *E*-value distribution of significant matches from the Nr as well as UniRef100 databases revealed that 69% of matched transcripts exhibited high levels of similarity (*E*-value < 10^−50^; Table S2).

Transcripts from the reference transcriptome assembly were also BLASTN analysed against CDSs from *M. truncatula*, chickpea and soybean (Tables S3–S5). The *M. truncatula* comparison revealed 65% of transcripts with matches, in which >96% of annotated transcripts exhibited hits to high confidence (HC) coding sequences. In total, BLASTN searches identified 41,693 (71%) transcripts with significant similarity to any of the comparator reference species, while 23,671 (57%) transcripts were found to have common matches between all three reference species. Other transcripts were either common between any two of the three references, or specific to each reference (11% to *M. truncatula*, 5% to chickpea and 1% to soybean).

There were 46,372 (79%) transcripts with common sequences in at least one of the databases (Nr, UniRef100, *M. truncatula*, chickpea and soybean). A total of 22,621 (48.8%) transcripts were found to have common matches between all databases (Figure 1). The percentage of transcripts with significant sequence similarity matches to the above-mentioned databases was higher for longer transcripts than shorter (Figure S1). For example, 99% of the transcripts longer than 1100 bp displayed significant matches to the databases. However, 21% (12,622) of the generated reference transcripts remained uncharacterised. The size of the uncharacterised transcripts ranged from 241 to 6372, with 65% (8176) of those transcripts less than 500 bp, and 90% (11,398) transcripts < 1000 bp.

**Figure 2:**
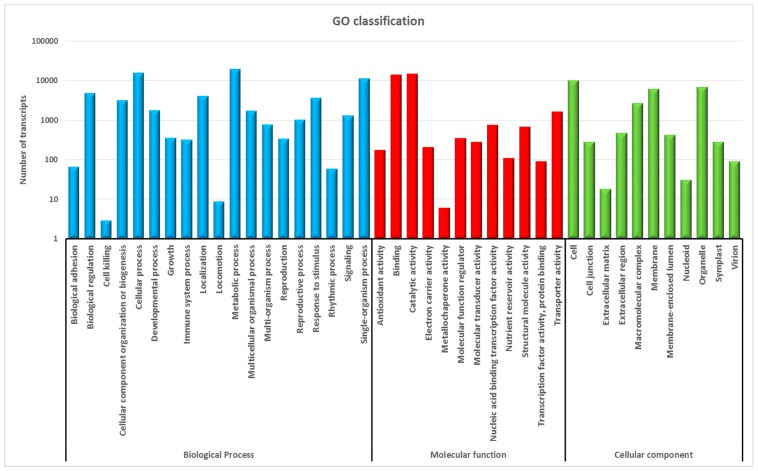
Functional annotation of assembled Cassab-derived reference transcripts based on gene ontology (GO) categorisation: GO analysis was performed at the level 2 for three main categories (biological process, molecular function, cellular component).

Gene Ontology (GO) terms were assigned based on the sequence similarity to Nr and InterPro databases, with 49% of transcripts (28,765) receiving at least one GO term. Within this group, assignments to the biological process category was highest with 54%, followed by molecular function category (25%) and the cellular component category (21%; Figure 2). Among the biological process categories, metabolic process (28%) and cellular process (23%) were prominently represented (Figure 2, Figure S2) indicating that tissues used in this study were undergoing extensive metabolic activity. A moderate number of transcripts were also involved in the single-organism process (16.3%), response to stimulus (5.2%) and developmental process (2.5%) categories, while only a limited number were associated with biological adhesion (0.09%), locomotion (0.01%) and cell killing (0.004%). Under the molecular function category, catalytic activity (45%) and binding (42%) were the most common (Figure 2, Figure S2). For the cellular component category, the majority of the transcripts were assigned to the cell (37%), organelle (25%) and membrane (22%) categories, while only 139 transcripts in total were assigned to virion (0.33%), nucleoid (0.11%) and extracellular matrix (0.06%) (Figure 2, Figure S2).

To further characterise the assembled transcripts and identify active biological processes, the Kyoto Encyclopedia of Genes and Genomes (KEGG) pathway database was analysed using eudicot species as references. Out of the 58,994 transcripts, 18,132 (31%) exhibited significant matches to 132 KEGG pathways corresponding to five modules; metabolism, cellular processes, genetic information processing, environmental information processing and organismal systems (Table S6). Metabolism (1444) was the highest represented module, followed by genetic information processing (910). In the metabolism module, metabolic pathways (810) was the most prevalent category, followed by biosynthesis of secondary metabolites (388), carbohydrate metabolism (338) and amino acid metabolism. Furthermore, mapping of transcripts against multiple pathways (such as glycolysis/gluconeogenesis pathway, nitrogen metabolism, flavonoid biosynthesis pathway and isoflavonoid biosynthesis pathway) revealed that all known genes involved in those pathways were represented.

The Cassab-derived assembled transcripts were also compared to two previously published lentil transcriptome sets [8,17] and the analysis revealed that the current assembly captured 90%–96% of transcripts from those assemblies (Table 2, Table S7). Approximately 55% of the Cassab reference transcripts were present in the three datasets, while 21% of the transcripts were exclusive to the current assembly (Table S7). Moreover, the N50 and average contig length of the Cassab reference assembly is higher than previous assemblies (Table 2). Transcripts from previous assemblies with no significant matches to the Cassab-derived reference unigene transcriptome were further examined, based on BLAST analysis to the *M. truncatula* and Nr databases, and ca. 99% of those transcripts failed to match any known plant sequence. BLASTN analysis of the Cassab reference transcriptome revealed that 58,835 (99.7%) transcripts displayed significant sequence similarity to lentil draft genome v0.8 (Table S7). The remaining 159 (0.3%) transcripts were further examined based on previous BLAST results (against Nr, UniRef100, *M. truncatula*, chickpea and soybean datasets). This process identified 86% (130) of these transcripts as being similar to plant-derived sequences, the majority (75%) being involved in plant growth and development processes, a small proportion (8%) being associated with stress-related responses, while the remainder encoded hypothetical or uncharacterised proteins.

### 2.3. Tissue-Specific Expression Analysis

**Figure 3:**
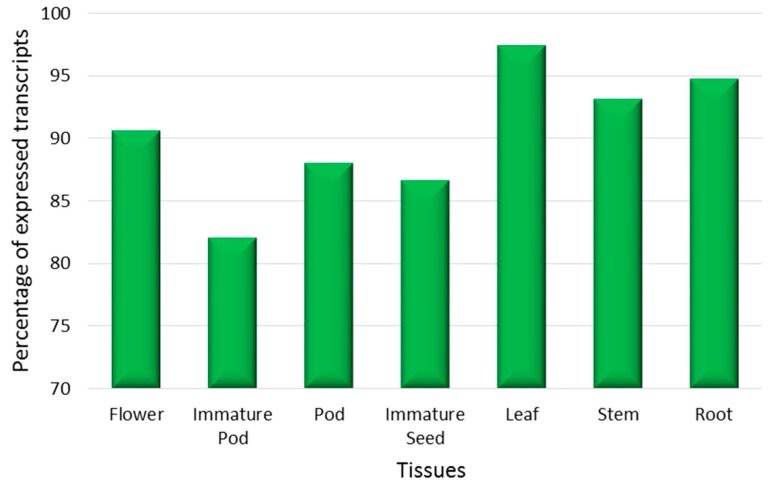
Expression patterns in different tissue samples: The percentage of transcripts expressed in each tissue sample.

In order to analyse the global expression of the reference unigene set, as well as validate the assembly, sequence reads from the individual libraries were aligned to the assembled Cassab reference unigene transcriptome. A similar number of transcripts (c. 51,000–57,000) were expressed in the majority of tissues, with the exception of immature pod for which a relatively lower number (48,000) was observed (Figure 3). Detailed transcript expression lists for each tissue are provided in Table S8. A majority (91.7%) of transcripts were common between the three tissue-type groups.

Analysis of tissue-specific expression revealed that ca. 98% of transcripts were present in more than one tissue, and 75% of the total transcripts were expressed in all tissues. However, the level of expression of common transcripts varied substantially between tissues. For example, pods expressed higher levels of transcripts corresponding to storage proteins such as vicillin, convicilin and embryonic abundant protein. Similarly, in leaf tissue the expression of ribulose bisphosphate carboxylase/oxygenase activase and light-harvesting chlorophyll-a/b binding protein genes was enriched. Tissue-specific expression was identified for a small proportion of transcripts (0.003%–1.8% per tissue, 1148 in total), the majority being associated with leaf (1.8%). Approximately 12% of the leaf specific transcripts lacked annotations, and among those annotated, 20% were classified as hypothetical or uncharacterised proteins, with a small proportion associated with plant defence, storage compartment (vacuolar protein sorting) and membrane transport.

**Figure 4:**
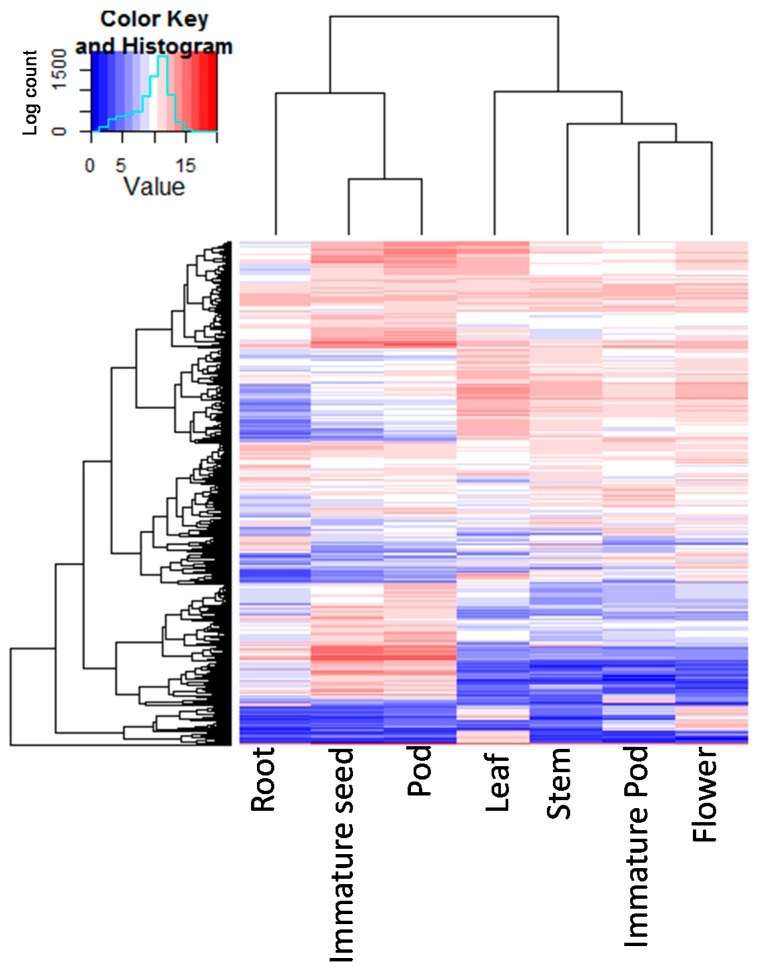
A heat map of the 1000 most differently expressed transcripts showing the hierarchical clustering of different tissues: The colour key represents the normalised log transformed counts. Red indicates high expression, white indicates intermediate expression and blue indicates low expression.

To demonstrate the value of the reference transcriptome assembly for gene expression analysis, transcription of two gene families (encoding embryogenesis-related protein and chlorophyll-a/b binding protein), were analysed in detail in the three tissue-type groups. The heat map of expression levels of transcripts associated with embryogenesis-related protein clearly separated the reproductive development-specific tissue cluster from the other two tissue types (Table S9). Similarly, chlorophyll-a/b binding protein expression analysis distinguished the gene expression of aerial tissues from subterranean tissue (Table S9). A heat map of the normalized transcription count from the top 1000 differently expressed transcripts was generated (Figure 4). Tissue-specific patterns (based on the annotations of the transcripts) were readily apparent in these data, with clustering by sample type (Figure 4, Table S9). Expression profiles revealed similarities among leaf, stem, immature pod and flower tissues, while root, pod, and immature seed tissues diverged into a separate cluster.

Validation of selected differentially expressed transcripts (16) was performed by quantitative reverse transcription polymerase chain reaction (qRT-PCR) analysis using a range of tissues including leaf, stem, root, flower, immature pod and immature seed. Levels of expression was evaluated for transcripts associated with a range of functions including chalcone reductase, carbonic anhydrase, ribulose bisphosphate carboxylase, convicilin, seed maturation protein and sugar transport proteins. A high proportion of the transcripts (15 out of 16) showed close to perfect concordance with the results from the RNA-Seq experiment (correlation coefficient of 0.9892; Table S10). However, a single transcript (Lc_contig_37524) displayed discordant outcomes (Table S10) for expression in leaf and immature seed tissues.

### 2.4. Identification of Candidate Genes for Tolerance to Boron Toxicity and Flowering Time in the Reference Transcriptome

To evaluate the degree of completeness, and exemplify the value of the transcriptome for interpretation of biological processes and development of tools for molecular breeding, candidate genes associated with both B toxicity tolerance mechanisms and flowering time variation were identified, based on comparison with known genes from other plant species. A text-based search of BLAST analysis data (against *M. truncatula* CDSs, Nr and UniRef100) identified a total of 57 candidate sequences (Table S11) to be associated with B toxicity tolerance in lentil. Approximately 70% (34 from a total of 49 genes) of known *M. truncatula* genes associated with B toxicity tolerance were identified in the current dataset corresponding to 50 lentil transcripts. Based on UniRef100 and Nr BLAST annotations, an additional 7 transcripts annotated as B transporters or major intrinsic proteins (MIPs) in other legume species were identified (Table S11).

**Figure 5:**
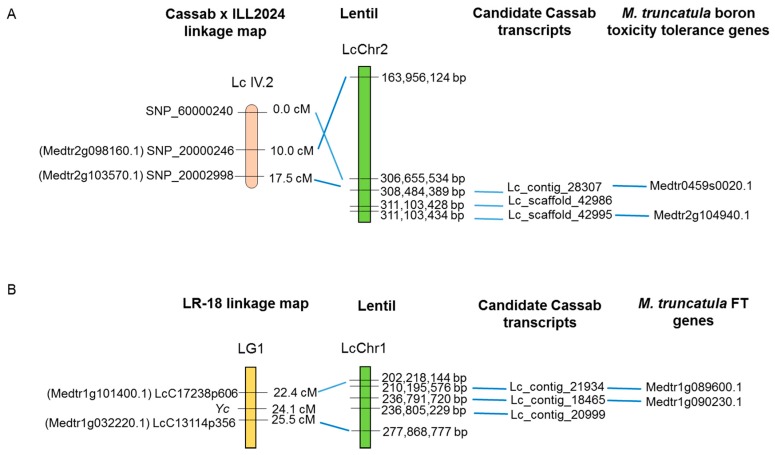
Schematic depictions of comparisons between (**A**) the boron tolerance quantitative trait loci (QTL)-containing-interval on linkage group (LG) Lc IV.2 of the Cassab × ILL2024 linkage map and both lentil genome assembly v1.2 and candidate Cassab transcripts with corresponding *M. truncatula* boron tolerance candidate gene sequences; (**B**) The flowering time QTL-containing interval on LG1 of the LR-18 linkage map and both lentil genome assembly v1.2 and candidate Cassab-derived transcripts with corresponding flowering time genes from *M. truncatula*. LGs or pseudomolecules are labelled accordingly. The names of genetic markers are shown to the left of each LG.

A BLASTN similarity search of the 57 boron tolerance-related transcripts to the draft lentil genome assembly v1.2 [13] revealed matches on different lentil pseudomolecules, most commonly on LcChr4, and least on LcChr7 (Table S11). Sequences underlying the genetic markers flanking the major B tolerance QTL (SNP_60000240, SNP_20000246 and SNP_20002998) on the Cassab × ILL2024 linkage map [38], as well as the corresponding *M. truncatula* genes (SNP_20000246-Medtr2g098160.1 and SNP_20002998-Medtr2g103570.1) matched genomic regions on LcChr2 (Figure 5). From the current transcriptome assembly a total of three transcripts (Lc_contig_28307, Lc_scaffold_42986 and Lc_scaffold_42995) with annotations as boron transporter-like protein and MIP genes obtained matches in same the genomic regions on lentil genome assembly v1.2. Furthermore, two *M. truncatula* MIP genes also showed matches to the same genomic region on LcChr2 (Figure 5).

A text-based search of the data from BLAST analysis of the reference transcriptome against *M. truncatula* CDSs, Nr and UniRef100 identified a total of 75 transcripts (66 genes) with high confidence related to processes of flowering (Table S11). Similarity searches of these transcripts against the draft lentil genome assembly v1.2 [13] revealed matches for 74 (99%) transcripts. A total of 40 (64%) *M. truncatula* genes associated with flowering time were identified in the reference transcriptome, exhibiting matches to 52 transcripts (from the total of 75 transcripts). The sequences of flanking SNP markers (LcC17238p606 and LcC13114p356) in the vicinity of the QTL region located on LG1 of the LR-18 RIL population-based map [39] identified matches on LcChr1 (Figure 5). Three Cassab-derived transcripts (Lc_contig_21934, Lc_contig_18465 and Lc_contig_20999) annotated as flower proteins obtained matches in the same genomic region on LcChr1 (Figure 5). Moreover, two *M. truncatula* FT-associated genes (Medtr1g089600.1 and Medtr1g090230.1) also revealed matches to the same genomic region on LcChr1 (Figure 5).

## 3. Discussion

### 3.1. Reference Transcriptome Assembly Characteristics

Lentil is a global food crop that is increasing in importance globally, especially in developing countries (including the Indian sub-continent and Middle to East Asia) due to nutritional value and inexpensive provision of dietary protein. Despite the global agronomic importance of lentil, scant genomic and genetic resources for crop improvement have until recently been available, limiting the application of marker-assisted selection strategies in breeding [8,17,43]. Generation and characterisation of a well-structured reference transcriptome provides an attractive means to redress this balance, as well as supporting completion of the current international sequencing effort.

The Australian cultivar of lentil, Cassab, was chosen for development of a reference transcriptome assembly. RNA from a broad range of aerial and subterranean tissues collected at various developmental stages was deeply sequenced to ensure representation of even lowly expressed tissue-specific transcripts. The final assembly resulted in 58,986 transcripts with total assembled length of 66,763,413 bp, which is highly comparable to that for *M. truncatula* (66,028,174 bp) [28] and soybean (68,278,578 bp) [44,45], but much higher than for chickpea (32,973,966 bp) [46,47]. The only relevant chickpea dataset available for use in this study involved gene models rather than an actual transcriptome dataset, and so the expressed portion of the genome in comparison to known protein-coding genes is likely to be under-represented. The N50 value of the Cassab reference transcriptome is longer than those of *M. truncatula* and chickpea (1506 bp), although the average coding sequence lengths were highly comparable (*M. truncatula*—1060 bp and chickpea—1166 bp) [28,46,47]. The higher N50 value probably be due to the presence of UTRs. The length of UTRs in different plant species varies from 291 bp (for sorghum) to 936 bp (rice *Oryza sativa* L.; [29]). The number of transcripts generated in the current study is comparable to the assembly of [8] (50,146 contigs), but with a significantly longer average gene length (N50 = 530 bp and average contig length = 501 bp) indicating a more complete assembly of the identified genes. When compared to the transcriptome assembly described by [17], the present study has identified fewer unique transcripts, but those assembled are significantly longer (15,354 contigs and 68,715 singletons; N50 = 349 bp and average contig length = 360 bp, respectively in the earlier study). In addition, comparison of the Cassab-derived reference transcriptome assembly to those already published indicated that for both of those studies, only a partial set of transcripts was represented (76% in the study of [17] and 59% in [8]). However, 96% of the transcripts were found to be common in reciprocal BLAST searches, indicating that the previously published lentil assemblies are fragmented in nature. This discrepancy may be due to the use of different sequencing technologies by the earlier studies, when compared to the present study. Both previous studies used 454 Roche pyrosequencing that is known as to be more error-prone than Illumina-based chemistry and so likely to result in fragmented assemblies [48]. The assembly statistics for the current lentil assembly are highly comparable to those of a recently published field pea transcriptome atlas (46,099 contigs with N50 length of 1667 bp) generated through use of the same technology [22]. The main objectives of the two previously published transcriptome studies was SNP discovery for linkage mapping and trait dissection purposes, and as a result, limited emphasis was made on varieties of tissue type, and depth of sequencing. The improved characteristics of the most recent lentil and field pea transcriptome assemblies reflect the superior performance of more contemporary sequencing platforms, with consequent higher value for functional genomics and molecular breeding applications.

### 3.2. Annotation of Gene Sequences

A total of ca. 71% of the transcripts were annotated by comparison with the NCBI Nr database, representing 27,396 unigenes. This number is comparable to the total number of genes identified in the genome sequences of other plant species such as *Arabidopsis thaliana* (25,498 protein-coding genes; Arabidopsis Genome Initiative 2000; [49]) and chickpea (28,269 gene models; [47]). However, a higher number of genes were identified in *M. truncatula* (62,388; [28]), soybean (46,430; [45]) and pigeon pea (48,690; [50]). Furthermore, the largest number of BLASTX matches was to *M. truncatula*, followed by chickpea and then other plant species, in agreement with the taxonomic relationships between these species. Within the sub-family Papilionoideae of the Fabaceae, lentil is most closely related to *M. truncatula* (both belonging to the Vicieae tribe), while chickpea belongs to the more distantly related Cicereae tribe within the Galegoid clade [51]. Comparison of the lentil transcriptome reference assembly to those of other closely related legume species (chickpea and field pea) revealed comparable results [4,47]. A total of 79% of transcripts exhibited matches when compared to all databases, including UniRef100, with a high proportion of transcripts being >1000 bp in length, indicating that smaller transcripts may represent alternative splice variants, as seen in similar studies [4]. Of the remaining 21% of transcripts that are uncharacterised, a further examination of the sub-set of transcripts >1000 bp in length indicated that ca. 80% contained open reading frames (ORFs) of ca. 300 bp (data not shown). These transcripts may represent pseudogenes, or repetitive elements, or genes with disrupted function. The percentage of transcripts that displayed similarity to known plant sequences is highly comparable to results from a previous study [32]. Comparison of the Cassab reference transcriptome assembly to previously published transcriptomes [8,17] indicated that up to 96% of the transcripts were represented in the current assembly, while the remaining 4% were uncharacterised. These transcripts may represent short fragments of genes that have not been properly assembled due to technical factors, or rarely expressed genes specific to the cultivars sequenced in those studies. These anomalies were further resolved by comparison of the current dataset with the lentil draft genome, revealing matches for 99.7% of transcripts. The remaining unmatched transcripts were still identified as plant-based proteins based on BLASTX results from Nr and UniRef databases, and could hence represent cultivar-specific components of the Cassab transcriptome. The KEGG analysis generated results similar to previous studies of field pea, in which 37% of transcripts were annotated by assignment to 157 pathways [4], as well as *M. truncatula*, for which 29.5% of genes were annotated.

### 3.3. Assessment of Tissue-Specific Gene Expression

Tissue-specific expression analysis indicated that similar numbers of transcripts were expressed in most of the tissues, with the exception of immature pod, for which a relatively lower number of transcripts was observed. This may be partly due to the relatively lower depth to which this tissue was sequenced (20 million reads) as compared to other tissues (109 million reads), which could have compromised the detection of low-abundance transcripts. The majority of transcripts were present in more than one tissue and common to all tissues, albeit with varying expression levels in each tissue. This observation provides confidence that the number of sampled tissue types was sufficient to support a high confidence reference transcript assembly. The preferential expression of transcripts in specific tissues identified the largest number (1.8%) in leaf, also observed in sorghum [52]. Identities of some of the tissue-specific transcripts were clearly related to a particular organ function. For example, in leaf a small proportion of annotated transcripts (<4%) was found to be related to compartmentalisation of solutes, but much larger proportions were classified as uncharacterised (20%) or non-annotated (12%). Leaf tissue expressed the largest proportion of transcripts within the overall assembly, suggesting that sampling from this source is a highly effective means for one-step partial gene-space identification.

Further assessment of the quality of the reference transcriptome revealed clear correlations between expression patterns and tissue-specific biological functions. For example, the chlorophyll-a/b binding protein exhibited a significant expression pattern in aerial tissues, while the reproductive tissue cluster can be clearly distinguished from subterranean tissue and vegetative tissues for the embryogenesis-related protein transcript profile, confirming validity of the data. The heat map of the 1000 most differently expressed transcripts revealed the functional relations between different tissues through clustering into two separate groups of root and seed tissues, and other aerial tissues, respectively. These results are consistent with knowledge of developmental biology, as a mature pod is part of the seed development pathway, while immature pod-shells and flowers are modified leaves [53]. Similar findings were obtained from the pea gene expression atlas, in which transcripts from nodule, root and seeds were well separated in a principal component analysis (PCA; [22]) from transcripts derived from above-ground vegetative tissues. The validation results using qRT-PCR for majority of the selected set of transcripts were highly consistent with the expression patterns identified by RNA-Seq method. However, for Lc_contig_37524, an inconsistent pattern was observed, which could be due to lack of specificity of the relevant primer pairs, so resulting in non-specific amplification, or detection of the expression profile from a paralogous gene sequence. In summary, these validation results reinforce the accuracy of Cassab reference unigen-based transcriptome profiling, and confirm that this database represents a comprehensive resource for transcript detection and accumulation.

### 3.4. Identification of Candidate Genes for Tolerance to B Toxicity and Flowering Time in the Reference Transcriptome

Previous studies in *A. thaliana* and *M. truncatula* have identified genes for efflux-type B transporters and members of the MIP family as controlling B toxicity tolerance [54,55,56,57]. The majority of known B tolerance-related genes described in model legume *M. truncatula* were shown to be present in the Cassab-derived transcriptome, validating integrated approach for large-scale identification of candidate genes for a given biological process. Candidate transcripts identified from lentil transcriptome assembly as well as candidate genes from *M. truncatula* based on flanking markers for B toxicity tolerance obtained matches on LcChr2. However, one of the QTL-flanking markers obtained matches on LcChr2 at a location distant to that of the other two SNP loci. This anomaly may be due to the ambiguity of marker ordering during the linkage mapping process or errors in assembly of the lentil genome, which is still in a preliminary form.

Genetic variation controlling the onset of flowering has important implications for performance of crop species. In legumes, several homologues of flowering gene known from model species have been extensively characterised [58,59]. A substantial number of lentil transcripts associated with flowering time were identified in this study, enhancing the potential to predict candidate genes for this trait. BLAST analysis for genes associated with each of these exemplar traits permitted substantial refinements of the candidate gene list in the QTL-containing regions. This information may be used for the identification of candidate genes in different lentil cultivars, and associated polymorphisms may be used to develop diagnostics for marker-assisted breeding studies.

## 4. Experimental Section

### 4.1. Plant Materials

Lentil plants (cv. Cassab, pedigree: ILL5690 × Digger [ILL0883/ILL0470]) were grown in a glasshouse in standard potting mix in 200 mm plastic pots at 22 ± 2 °C with a photoperiod of 16/8-h (light/dark). Leaf and stem tissues from multiple nodes, as well as root tissues were collected from 4 weeks-old plants (three replicates). Fully open flowers, immature pods (8–12 days after flowering), pods and immature seeds (18–23 days after flowering) were collected in three replicates at the appropriate time points. The sampled tissues were immediately frozen in liquid nitrogen before storage at −80 °C. Prior to RNA extraction, samples from the replicates for each tissue were pooled in equal quantities. Total RNA was extracted using the RNeasy^®^ Plant Mini Kit (QIAGEN, Hilden, Germany) following manufacturer’s instructions. The concentration of RNA was confirmed using a spectrophotometer (Thermo-Scientific, Wilmington, DE, USA) at the wavelength ratios of A260/230 and A260/280 nm. The integrity of total RNA was determined by electrophoretic separation on 1.1% (*w*/*v*) denaturing agarose gels.

### 4.2. Library Preparation

RNA-Seq libraries with an approximate insert size of 350 bp were generated using the SureSelect Strand Specific RNA Library Prep Kit and evaluated using the TapeStation 2200 platform with D1000 ScreenTape System (Agilent Technologies, Santa Clara, CA, USA) according to the manufacturer’s protocols. Each RNA-Seq library was generated with a unique barcode and an equal mass of each sequencing library was combined to create a single pooled sample for sequencing. The pooled sample was quantified using the KAPA library quantification kit (KAPA Biosystems, Boston, MA, USA). Libraries were pair-end sequenced using the HiSeq 2000 system (Illumina Inc., San Diego, CA, USA).

### 4.3. De Novo Transcriptome Sequence Assembly

Following fastq data generation, the raw sequence reads were filtered using a custom perl script and Cutadapt v1.4.1 [60]. Reads were filtered by removing adaptor sequences along with reads and bases of low quality (containing more than 10% bases with *Q* ≤ 20). Reads with 3 consecutive unassigned nucleotides (N) were trimmed, as were reads with more than 3 consecutive nucleotides showing a phred score of ≤20. Finally, reads shorter than 50 bp in length were removed from the final set. The remaining high quality sequence reads were then de novo assembled using SOAPdenovo-Trans [61] with *k*-mer size of 91. Fork, bubble and complex loci from the SOAPdenovo-Trans assembly were further combined using the CAP3 assembler [62], with 95% identity and a minimum of 50 bp overlap to produce longer, more complete consensus sequences. Finally, transcripts shorter than 240 bp were discarded, being less than the length of a single pair of sequence reads.

### 4.4. Functional Annotation and Classification of Reference Transcriptome

The assembled Cassab reference transcripts were BLASTX analysed [63] against the Nr protein database maintained by NCBI (as of 12-05-2015) and the UniRef100 database version 1.0 under the threshold parameter of *E*-value < 10^−10^. For further assembly annotation, similarity searches of the transcripts were performed against the CDSs of *M. truncatula* v4.0 (*Medicago truncatula* Genome Project [28]), chickpea (International Chickpea Genetics and Genomics Consortium [46]) and soybean (PlantGDB [44]) using BLASTN with a threshold *E*-value of < 10^−10^. In the reference *M. truncatula* v4.0 CDS dataset, gene models are classified into high and low confidence classes (HC and LC) based on the different levels of EST/RNA-seq/protein alignments and homology [28]. In those instances in which conflicting highest BLAST annotations between different taxonomic kingdoms were generated from the BLASTX and BLASTN analyses, the highest match from the BLAST analyses was identified based on the criteria of “higher % similarity” and “lower *E*-value”.

The assembled transcripts were compared to the KEGG database based on BLASTX queries. The KEGG pathway annotation was performed in the KEGG Automatic Annotation Server (KAAS; [64,65]) to further characterise the assembly. In addition, the assembled transcripts were evaluated using GO terms, via the Blast2GO PRO software program [66], used with an *E*-value threshold cut-off of < 10^−10^ based on Nr and InterPro annotations. To validate the current assembly, unigene sequences from previous lentil transcriptome sequencing studies [8,17], as well as genomic sequences from the draft lentil genome assembly v0.8 [12,13] were BLASTN analysed against the transcriptome dataset generated in the current study with an *E*-value < 10^−10^.

### 4.5. Tissue-Specific Expression Analysis

To obtain tissue-specific gene expression data, the trimmed sequence reads generated from each of the RNA-Seq libraries were reference aligned against unigenes using the BWA-MEM software package using default settings [67]. As the individual libraries varied in terms of generated sequence reads, relative expression based on read counts that had been normalised on the 75^th^ percentile were used for this purpose as previously described [4]. Data from individual tissues were analysed, as well as being grouped into three major groups of tissue type: reproductive stage-specific tissues (flower, immature pod, pod and immature seed); subterranean tissue (root); and vegetative tissues (leaf and stem). The normalised read counts were combined from each individual tissue for this purpose and then the expression profile for each group was analysed.

To validate the reference transcriptome dataset, the relative level of expression for embryogenesis-related protein and chlorophyll-a/b binding protein gene family members in each tissue type group was determined by analysis of the normalised read count dataset. The respective heat maps were generated using the software package R with the heatmap.2 function from the gplots CRAN library. Further assessment of the reference transcriptome was performed based on the profiles of the 1000 most highly differently expressed transcripts (selected based on the highest variance among different tissues from the normalised read count dataset described above). A heat map of the 1000 differentially expressed transcripts was generated as described above.

The expression of a selected set of differentially expressed transcripts (16) identified by the RNA-Seq analysis was re-examined through qRT-PCR analysis. RNA extraction from different tissues (leaf, stem, root, flower, immature pod and immature seed) was performed as detailed above and the total RNA was treated with DNase I (QIAGEN). Complementary DNA from total RNA (1 µg) was prepared using the QuantiTect^®^ Reverse Transcription Kit (QIAGEN) according to the manufacturer’s instructions. A no-reverse transcriptase control was included in the experiment for each tissue to check any amplification products due to the presence of genomic DNA. The glyceraldehyde-3-phosphate dehydrogenase (GAPDH) gene was used as an internal reference gene. The primer sequences (Table S12) for validation of the selected transcripts were designed using BatchPrimer3 [68], with default parameters for a product size of 100 to 120 bp and an optimum annealing temperature between 55 and 60 °C. The qRT-PCR was performed in CFX384 Touch™ Real-Time PCR Detection System (Bio-Rad Laboratories Inc., Hercules, CA, USA), and the reactions were performed in duplicate for each sample with a reaction volume of 12 μL containing 0.5 μM each primer, 1.0 μL of 1 in 10 diluted cDNA and 6 μL of FastStart DNA Master SYBR^®^ Green I (Roche Diagnostics, Mannheim, Germany). Each set of reactions included a negative control with no template. The thermal cycling conditions were 95 °C for 10 min followed by 45 cycles of 95 °C for 30 s, 54 °C for 30 s, 72 °C for 30 s [69]. The specificity of qRT-PCR primers was confirmed by melting curve analyses; range 54–95 °C, increasing the temperature in a stepwise fashion by 0.5 °C every 40 s. Normalisation of qRT-PCR data was achieved by subtracting the *C*q values of the internal reference gene from the *C*q values of the target genes to obtain ∆∆*C*q method [CFX Manager™ software version 3.1 (Bio-Rad Laboratories Inc., Hercules, CA, USA), [70]. The correlation between the RNA-Seq and qRT-PCR data was assessed by calculating the Pearson's correlation coefficient in Excel.

### 4.6. Identification of Candidate Genes for Tolerance to B Toxicity and Flowering Time in the Reference Transcriptome

To identify the candidate genes associated with tolerance to B toxicity [54,55,56,57] and time to flowering [58,59] in lentil, a text-based search of the Cassab transcriptome BLAST analysis data (against *M. truncatula* CDS, Nr and UniRef100) was performed. Two SNP-based linkage maps [38,39] which were used to precisely map QTLs for B toxicity tolerance and time to flowering were selected in this study. Previously, 4 QTLs (located on LG1, LG2, and LG7) associated with flowering time were identified using phenotypic data from multiple site-years [41], However, only the QTL on LG1 was significant and hence was used to identify candidate genes.

Sequences identified from the transcriptome, along with candidate genes related to B tolerance mechanism and time to flowering in *M. truncatula* v4.0 CDS and the sequences of the SNP markers flanking the QTL regions from previous studies [38,39] were BLASTN (threshold *E*-value of <10^−10^) analysed against the draft lentil genome assembly v1.2 [13] through the KnowPulse website [13] to identify their genomic locations. Lentil genome assembly v1.2 contains assembled pseudomolecules representing biological chromosomes as well as unanchored contigs and scaffolds.

## 5. Conclusions

The Cassab-derived reference unigene transcriptome dataset that has been generated in this study represents a valuable new resource for a range of applications. As well as providing information on gene content and expression in the complex lentil genome, the RNA-Seq approach established a robust genomic resource for subsequent applications. Comparison of the reference transcriptome to the lentil draft genome sequence assisted validation of results from the current study, indicating that this dataset will prove highly useful for subsequent applications in breeding and functional genomics. The dataset can be queried to identify patterns of gene expression associated with individual tissue types, and explored in future studies to gain insight into gene functions and hence biological functions relevant to key agronomic traits in this important grain legume species. In addition, the dataset will be valuable for annotation of future whole genome assemblies, and a high-quality reference transcriptome will permit detection of sequence polymorphisms, such as those generated by genotyping-by-sequencing (GBS) systems, with consequent impact on cultivar development.

## Figures and Tables

**Table 1:** Overview of sequencing outputs and assembly.

Primary Assembly	Statistics
SOAPdenovo-Trans	
Total number of filtered reads	660,842,789
Total number of reads in contig assembly	553,644,566
Total number of scaffolds and contigs	77,778
N50	1731
Total base pairs	76,992,636
Total base pairs without 'N'	75,665,777
Secondary assembly	
CAP3	
Total number of scaffolds and contigs	58,994
N50	1719
Total base pairs	66,767,914
Total base pairs without 'N'	65,746,675

**Table 2:** Overview of different assembly statistics and BLAST analysis.

Assembly	Number of Transcripts	N50	Average Transcript Length (bp)	Number of Transcripts with BLAST Hit to Reference Assembly	Number of Unique Reference Transcripts Having Hits
Kaur et al. (2011) assembly [17]	84,069	349	360	75,747	23,417
Sharpe et al. (2013) assembly [8]	50,146	530	501	48,013	16,905
Reference assembly	58,994	1719	1132	-	-

## Supplementary Materials

The data sets supporting the results of this article are included within the article and its Supplementary Materials. The sequence data has been deposited at DDBJ/EMBL/GenBank under the SRA accessions—SRR5002677-SRR5002683. BioProject ID for transcriptome is PRJNA352096. Supplementary materials can be found at www.mdpi.com/1422-0067/17/11/1887/s1.

## Abbreviations

B	Boron
bp	Base pair
BLAST	Basic Local Alignment Search Tool
cDNA	Complementary DNA
CDS	Coding DNA sequences
CO	CONSTANS
DNA	Deoxyribonucleic acid
EST	Expressed sequence tag
FT	Flowering time
GAPDH	Glyceraldehyde-3-phosphate dehydrogenase
Gbp	Giga base pair
GBS	Genotyping-by-sequencing
GO	Gene Ontology
HC	High confidence
KAAS	KEGG Automatic Annotation Server
KEGG	Kyoto Encyclopedia of Genes and Genomes
LOD	Logarithm (base 10) of odds
Mb	Mega base pair
MIP	Major intrinsic protein
mRNA	Messenger RNA
NCBI	National Center for Biotechnology Information
NGS	Next-generation sequencing
Nr	Non-redundant
ORF	Open reading frame
PCA	Principal component analysis
qRT-PCR	Quantitative reverse transcription polymerase chain reaction
RNA	Ribonucleic acid
RNA-Seq	RNA sequencing technology
UTRs	Untranslated regions
W/V	Weight/volume
