# Supplementary material for: Generation and Characterisation of a Reference Transcriptome for Lentil (Lens culinaris Medik.)
Source: Int J Mol Sci. 2016 Nov 12;17(11):1887. doi: 10.3390/ijms17111887 (PMC5133886; doi:10.3390/ijms17111887)
Supplement: Supplementary file 1 [file ijms-17-01887-s001.zip › ijms-141799-Supplementary Material/ijms-141799-supplemeantray.pdf]

Supplementary Materials: Generation and Characterisation of a Reference Transcriptome for Lentil (*Lens culinaris* Medik.)

Shimna Sudheesh, Preeti Verma, John W. Forster, Noel O. I. Cogan and Sukhjiwan Kaur

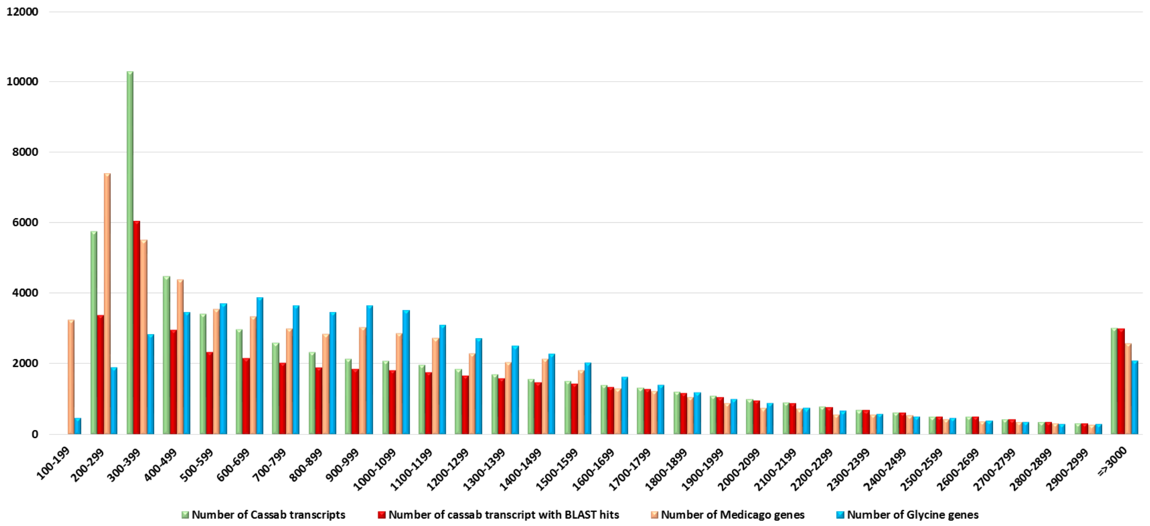

Figure S1. Length distribution of transcripts/genes—Cassab reference transcripts, Cassab reference transcripts with Nr BLAST hits, Medicago genes and Glycine genes.

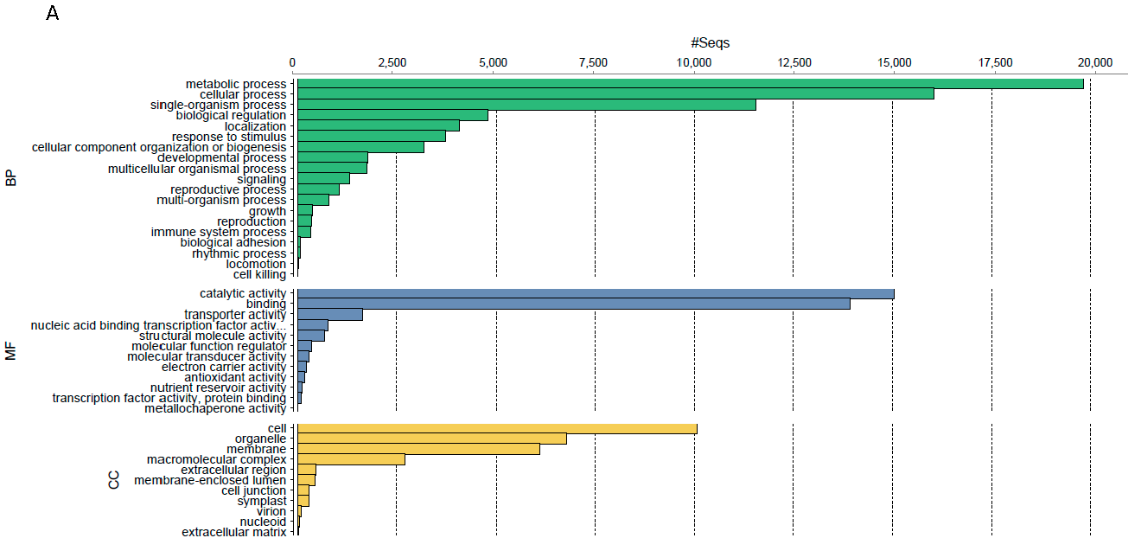

Figure S2. *Cont.*

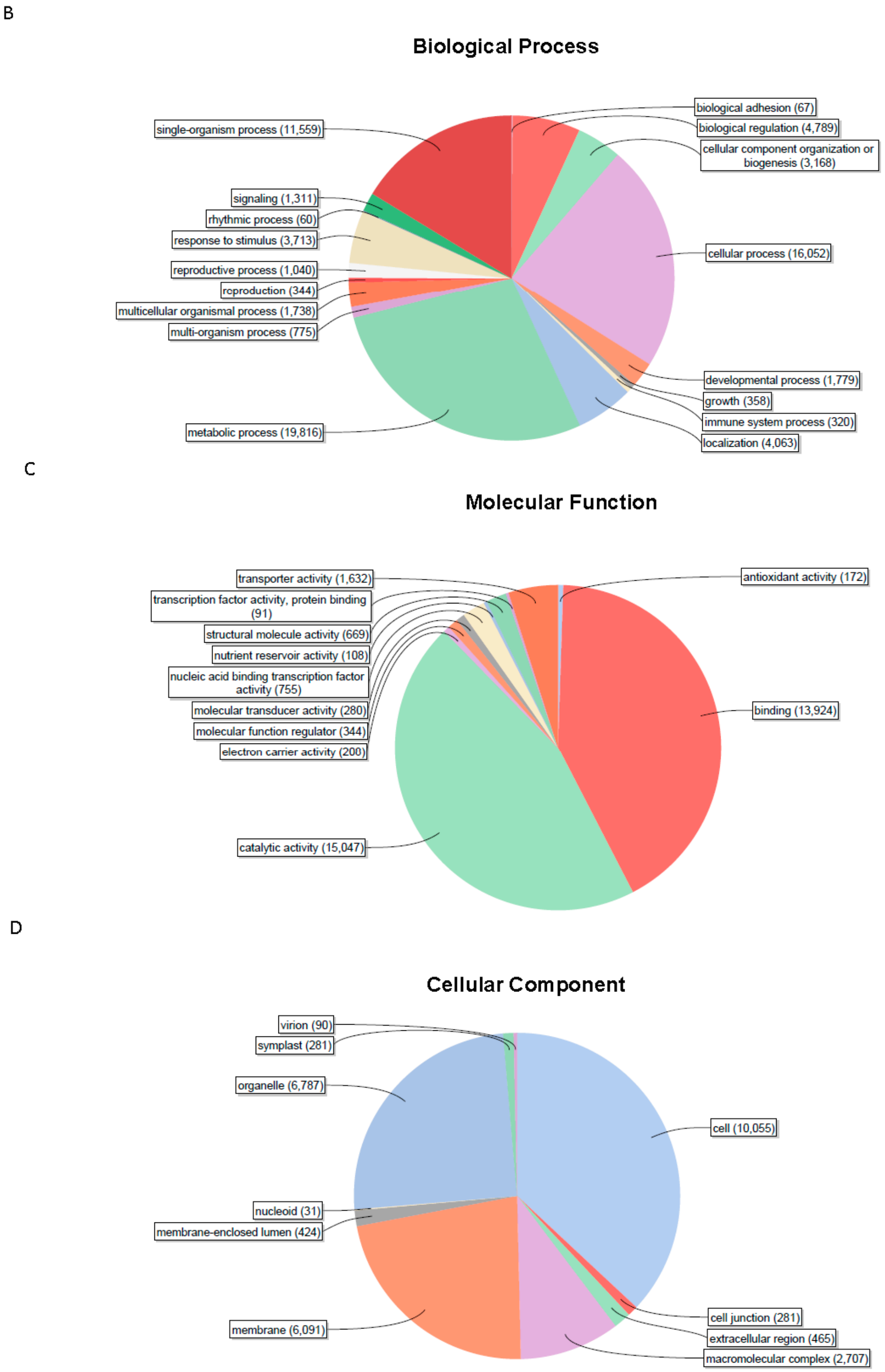

Figure S2. (**A**) Distribution of the annotated transcripts under gene ontology categories such as biological process (BP), molecular function (MF) and cellular component (CC); (**B**) Distribution of the annotated transcripts under gene ontology category–biological process (BP); (**C**) Distribution of the annotated transcripts under gene ontology category–molecular function (MF); (**D**) Distribution of the annotated transcripts under gene ontology category–cellular component (CC).
